# Differential Regulation of rRNA and tRNA Transcription from the rRNA-tRNA Composite Operon in *Escherichia coli*

**DOI:** 10.1371/journal.pone.0163057

**Published:** 2016-12-22

**Authors:** Hiraku Takada, Tomohiro Shimada, Debashish Dey, M. Zuhaib Quyyum, Masahiro Nakano, Akira Ishiguro, Hideji Yoshida, Kaneyoshi Yamamoto, Ranjan Sen, Akira Ishihama

**Affiliations:** 1 Research Center for Micro-Nano Technology, Hosei University, Koganei, Tokyo, Japan; 2 Laboratory for Chemistry and Life Science, Tokyo Institute of Technology, Nagatsuda, Yokohama, Japan; 3 Centre for DNA Fingerprinting and Diagnostics, Hyderabad, India; 4 Institute for Virus Research, Kyoto University, Sakyo-ku, Kyoto, Japan; 5 Department of Physics, Osaka Medical College, Takatsuki, Osaka, Japan; 6 Department of Frontier Bioscience, Hosei University, Koganei, Tokyo, Japan; Baylor College of Medicine, UNITED STATES

## Abstract

*Escherichia coli* contains seven rRNA operons, each consisting of the genes for three rRNAs (16S, 23S and 5S rRNA in this order) and one or two tRNA genes in the spacer between 16S and 23S rRNA genes and one or two tRNA genes in the 3’ proximal region. All of these rRNA and tRNA genes are transcribed from two promoters, P1 and P2, into single large precursors that are afterward processed to individual rRNAs and tRNAs by a set of RNases. In the course of Genomic SELEX screening of promoters recognized by RNA polymerase (RNAP) holoenzyme containing RpoD sigma, a strong binding site was identified within 16S rRNA gene in each of all seven rRNA operons. The binding *in vitro* of RNAP RpoD holoenzyme to an internal promoter, referred to the promoter of riRNA (an internal RNA of the rRNA operon), within each 16S rRNA gene was confirmed by gel shift assay and AFM observation. Using this riRNA promoter within the *rrnD o*peron as a representative, transcription *in vitro* was detected with use of the purified RpoD holoenzyme, confirming the presence of a constitutive promoter in this region. LacZ reporter assay indicated that this riRNA promoter is functional *in vivo*. The location of riRNA promoter *in vivo* as identified using a set of reporter plasmids agrees well with that identified *in vitro*. Based on transcription profile *in vitro* and Northern blot analysis *in vivo*, the majority of transcript initiated from this riRNA promoter was estimated to terminate near the beginning of 23S rRNA gene, indicating that riRNA leads to produce the spacer-coded tRNA. Under starved conditions, transcription of the rRNA operon is markedly repressed to reduce the intracellular level of ribosomes, but the levels of both riRNA and its processed tRNA^Glu^ stayed unaffected, implying that riRNA plays a role in the continued steady-state synthesis of tRNAs from the spacers of rRNA operons. We then propose that the tRNA genes organized within the spacers of rRNA-tRNA composite operons are expressed independent of rRNA synthesis under specific conditions where further synthesis of ribosomes is not needed.

## Introduction

**Fig 1:**
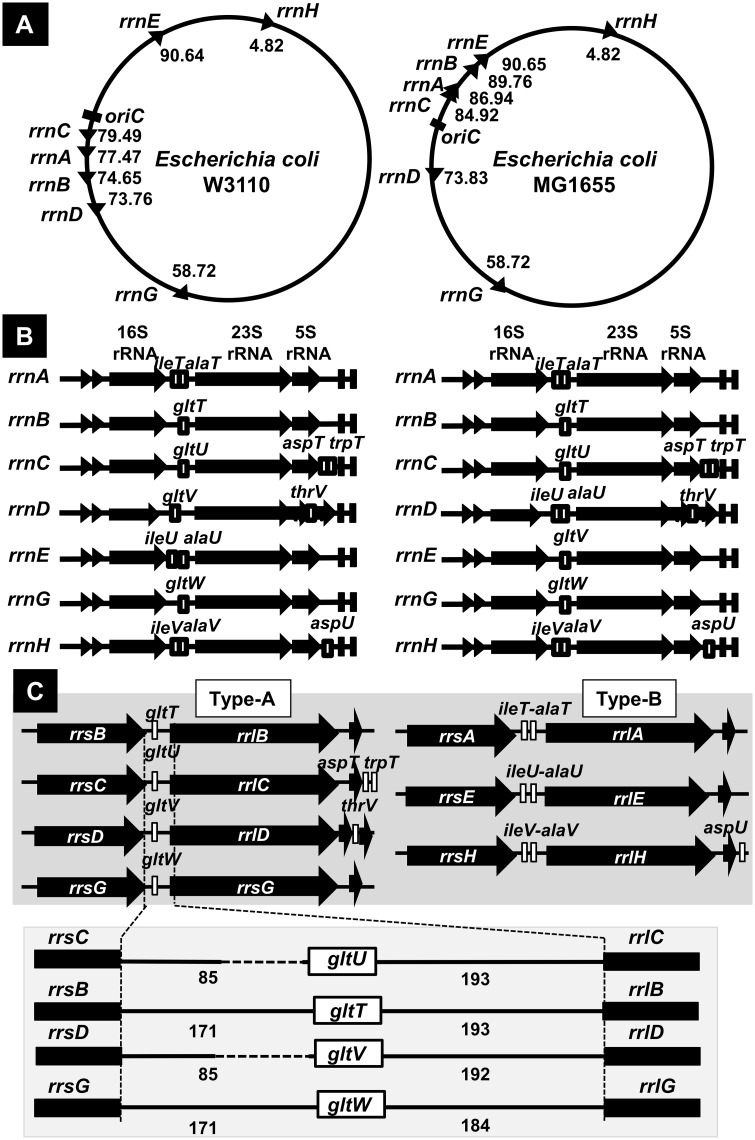
rRNA operons in *E*. *coli* K-12 W3110 and K-12 MG1655. [A] Both *E*. *coli* K-12 3110 and K-12 MG1655 strains carry seven *rrn* operons, but their locations on the genome are different in two strains because of a long-distance inversion in W3110 strain through homologous recombination between the *rrnD* and *rrnE* operons (see S1 Fig). [B] The gene organization of each *rrn* operon is essentially the same between W3110 and MG1655 strains except that the *rrnD* operon of W3110 corresponds to the *rrnE* operon of MG1655; and the *rrnE* operon of W3110 corresponds to the *rrnD* operon of MG1655. [C] Seven *rrn* operons can be classified into two types with respect to the tRNA gene(s) inside the spacer between 16S and 23S rRNA gene. In this study, we analyzed the riRNA gene within type-A *rrn* operons.

Ribosome, the core apparatus of translation, in *Escherichia coli* is composed of three species of rRNA (16S, 23S and 5S rRNAs) and a total of 55 species of ribosomal protein. rRNA plays fundamental roles as the structural and functional components of the ribosome. The genome of *E*. *coli* contains seven rRNA operons, each consisting of 16S, 23S, and 5S rRNA in this order. All seven rRNA operons contain, besides three rRNA genes, specific tRNA genes within the spacer between 16S and 23S rRNA genes and after 23S rRNA gene. The complete genome sequence of *E*. *coli* was first determined for two K-12 strains, MG1655 [1] and W3110 [2]. Both contain the same seven sets of *rrn* operons, but due to the inversion of a long segment of about 783 Kbp in length within the W3110 genome [3], the alignment of seven *rrn* operons is different between two well-characterized *E*. *coli* K-12 genomes (Fig 1A; also see S1 Fig). The levels of RNA polymerase (the core apparatus of transcription) and ribosomes (the core machinery of translation) correlate the rate of bacterial growth [4–12]. The presence of seven rRNA operons in *E*. *coli* might be needed for optimal adaptation to changing physiological conditions [13,14]. After systematic approaches of making *E*. *coli* strains with deletion of rRNA operons [13,15], the level of growth reduction was found to roughly correlate with the deleted number of rRNA operons. The presence of even a single rRNA operon on the genome is, however, able to produce as much as 56% of wild-type levels of rRNA supposedly through enhanced expression of the remaining rRNA operon [15]. The certain level of correlation between the number of *rrn* operons and the rate of cell growth was also confirmed by using a set of engineered rRNA opeon copy-number variants [16]. These findings also indicated that *E*. *coli* harbors an excessive level of ribosomes, keeping a considerable level of ribosome storage. In fact, unused ribosomes are stored in inactive forms by forming ribosome dimers after interaction with dimerization factors such as ribosome modulation factor RMF [17].

Seven *rrn* operons can be classified into two groups. Type-A *rrn* operons include tRNA^Glu^ gene within the intergenic spacer between 16S and 23S rRNA genes while type-B *rrn* operons contain two tRNA genes (tRNA^Ile^ and tRNA^Ala^) (Fig 1B). In the case of *E*. *coli* K-12 W3110 that was used in this study, the *rrnB*, *rrnC*, *rrnD* and *rrnG* operons belong to type-A while the *rrnA*, *rrnE* and *rrnH* operons belong to type-B (Fig 1B and 1C). Downstream of the 23S RNA genes, there are extra tRNA genes in three rRNA operons, *i*.*e*., *thrV* downstream of the *rrnD* operon and *aspU* downstream of the *rrnH* operon, and *aspT* and *trpT* downstream of the *rrnC* operon. These 3’-terminal tRNA genes appear to be transcribed from their own promoters [18]. To achieve the high growth rate, *E*. *coli* maximizes the synthesis rate of both rRNA and tRNA in a coordinate fashion. After determination of the promoter strength, seven rRNA operons were found to be differentially controlled depending on the nutrient conditions, raising a possibility of as yet unidentified differential roles between seven rRNA operons [19]. Differential control of seven rRNA operons might be attributable to the difference in tRNA species in each *rrn* operon or the different functional roles of rRNAs [note that about 2% sequence of rRNA is different between seven rRNA operons (S1 Table)].

Transcription of all seven *rrn* operons is initiated from tandem promoters (upstream strong P1 and downstream weak P2) in a control region upstream of the 16S rRNA gene [20,21]. The P1 promoter plays a major role in high-level expression of the rRNA operons in exponentially growing cells while P2 plays a role of low and basal level synthesis of rRNA. Transcription initiation from P1 promoter is under the positive control by Fis [22–24] and negative control by H-NS [25,26], both playing architectural and regulatory roles in the nucleoid, and two nucleotides, initiation nucleoside triphosphates (iNTPs) [9,12,27,28] and stringent control signal ppGpp (and pppGpp) [8,9,29,30]. Primary transcripts or precursor rRNAs are subsequently processed into mature 16S rRNA, individual tRNAs, 23S rRNA, and 5S rRNA by a set of processing nucleases [31,32].

In the course of mapping of the constitutive promoters that are recognized by RNA polymerase (RNAP) RpoD holoenzyme alone in the absence of supporting transcription factors, we found a strong RpoD holoenzyme-binding peak on the 16S rRNA gene [33,34]. Here we identified the RNAP RpoD holoenzyme-binding site within the 16S rRNA gene for each of all seven *rrn* operons. As an attempt to identify the physiological role(s) of these internal promoters within the 16S rRNA genes, we then analyzed the precise location of this strong internal promoter within the 16S rRNA gene in the *rrnD* operon of *E*. *coli* K-12 W3110, which was used as a representative of the type-A *rrn* operon, by using the gel shift assay with different DNA probes and the AFM observation. The promoter activity was then examined by an *in vitro* transcription assay and an *in vivo* reporter assay using the internal promoter within the 16S rRNA gene from the *rrnD* operon. Results altogether indicated that this internal promoter within the 16S rRNA gene was found to direct transcription of the spacer region between 16S and 23S rRNA, generating an internal RNA, referred to riRNA in this report. The synthesis of riRNA was further confirmed *in vivo* by Northern blot analysis. Since the activity of riRNA promoter increases, relative to the major promoters, in the stationary phase, we propose the possibility that transcription of riRNA takes place only when transcription from the major promoters of *rrn* operons become silent, allowing the synthesis of spacer tRNAs independent of rRNA synthesis.

## Results

### Identification of the constitutive RpoD promoters within all seven 16S rRNA genes

**Fig 2:**
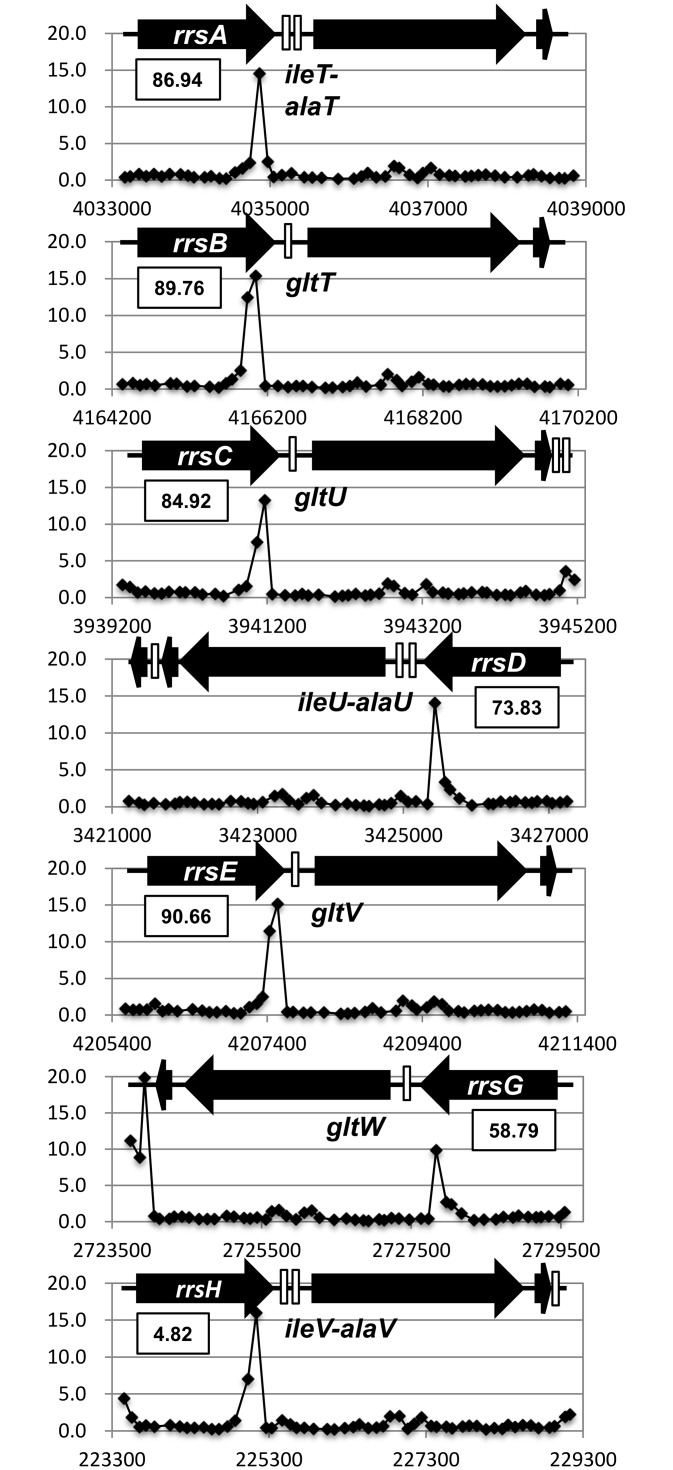
RNAP RpoD holoenzyme-binding sites within seven *rrn* operons. The binding sites of RNAP RpoD holoenzyme on the *E*. *coli* K-12 genome were identified using the Genetic SELEX screening system. One strong constitutive promoter that was recognized by the reconstituted RNAP RpoD holoenzme alone is located at 3’-terminal region of 16S rRNA gene in each of all seven *rrn* operons.

To identify the whole set of constitutive promoters under the direct control of RNAP RpoD holoenzyme alone in the absence of supporting regulatory factors, we performed the improved Genome SELEX screening [34,35]. RpoD holoenzyme was reconstituted from the sigma-free RNAP core enzyme and 4-fold molar excess of the purified RpoD sigma subunit [36]. Among the antibodies against each of the core enzyme subunits, the anti-RpoC antibody used in this study was the best with respect to the recovery of antigen-antibody complexes. After incubation with a mixture of genome DNA fragments of 200–300 bp in length, RNAP-DNA complexes were immuno-purified using anti-RpoC antibody. After four cycles of Genomic SELEX, DNA fragments bound with RpoD holoenzyme were purified and then subjected to DNA chip analysis using an *E*. *coli* tiling array. Based on the distribution of RpoD holoenzme-bound DNA fragments, a total of 2,701 RNAP RpoD holoenzyme-binding sites were identified, from which we predicted a total of maximum 669 constitutive promoters that are recognized by RNAP RpoD holoenzyme alone [34]. One surprising finding is that approximately 60% of the RpoD holoenzyme-binding sites thus identified were located within open reading frames. This finding indicates that a number of as yet uncharacterized promoter-like sequences exist inside genes. In the case of RNA genes, we also identified high-level binding of the RNAP RpoD holoenzyme within the 16S rRNA genes for all seven rRNA operons (Fig 2). Since these constitutive promoters exist essentially at the identical positions for all seven 16S rRNA genes of the *E*. *coli* genome K-12, we then decided to analyze a possible physiological role(s) of the riRNA promoter that is recognized by RNAP RpoD holoenzyme alone in the absence of other supporting factors.

### Binding *in vitro* of RNAP RpoD holoenzyme to riRNA promoter: Gel shift assay

In order to confirm the binding of RNAP RpoD holoenzyme to the riRNA promoter within the 16S rRNA gene, we first performed *in vitro* gel shift assay. Seven rRNA operons of *E*. *coli* K-12 W3110 can be classified into two groups: type-A containing a single tRNA^Glu^ gene inside the spacer between 16S and 23S rRNA genes; and type-B carrying two tRNA genes (tRNA^Ile^ and tRNA^Ala^) in this spacer (Fig 1C). As a representative riRNA promoter within the 16S rRNA genes, we focused on the *rrnD* operon for detailed analysis, but the sequence of the tRNA^Glu^ gene is identical for four type-A *rrn* operons (*rrnB*, *rrnC*, *rrnD* and *rrnG*). Furthermore the intragenic spacer sequences between the 16S rRNA and tRNA^Glu^ gene and between the tRNA^Glu^ and 23S rRNA genes are also highly conserved (S3 Fig). In most of the experiments noted below, we detected expression of all four type-A *rrn* operons.

**Fig 3:**
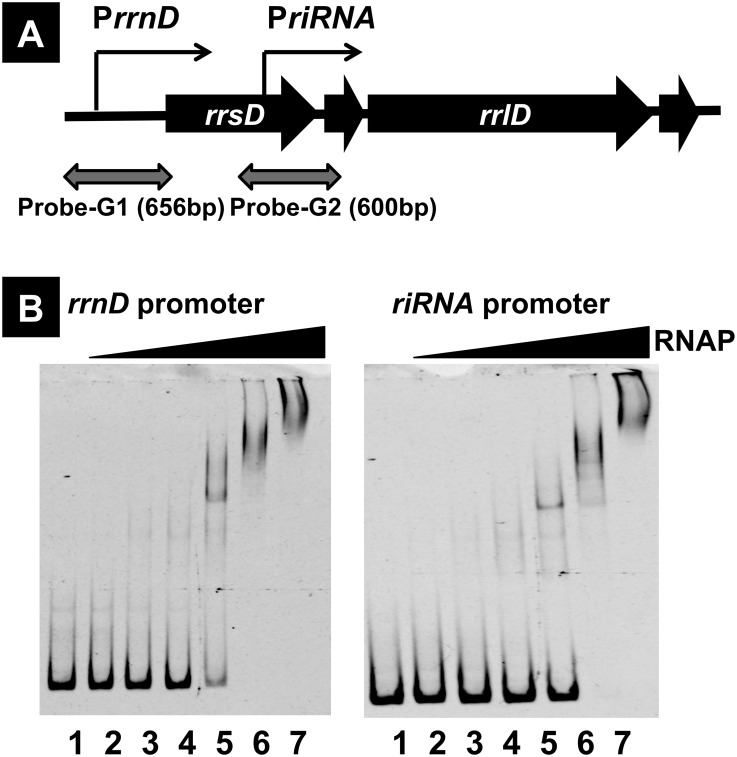
Gel shift assay of RNAP-DNA complexes. [A] The probes for the gel shift assay of RNAP-DNA complex formation was prepared using PCR-amplified promoter fragments of the main promoter (probe G1 in Table 1) and the riRNA promoter (probe G2 in Table 1) from the *rrnE* operon promoter. As a reference control probe (probe G3 in Table 1), we prepared a 3’-proximal segment of 23S rRNA gene. Primers used for PCR amplification are described in S2 Table. [B] The gel shift assay was performed using a fixed amount (50 nM each) of the promoter probes and the increased concentrations of reconstituted RpoD holoenzyme (lanes 1 to 7, 0, 3, 10, 30, 100, 300 and 1000 nM). The binding affinity of RpoD holoenzyme to the riRNA promoter (probe G2) was as high as that to the major *rrnE* operon promoter (probe G1). No binding of RNAP was detected with the reference probe G3.

A DNA probe of 600 bp in length including the predicted riRNA promoter of the *rrsD* gene was prepared by PCR amplification (Fig 3A and Table 1, probe G1) and subjected to the gel shift assay in parallel with the 656 bp-long probe containing the major promoters, P1 and P2, of the same *rrnD* operon (Fig 3A and Table 1, probe G2). Protein concentration-dependent increase of RNAP RpoD holoenzyme binding was observed with the riRNA probe (Fig 3B). As a reference, we also carried out the gel shift assay using 554 bp-long probe G3 corresponding to 3’-proximal region of 23S rRNA (Fig 3A, probe G3). Virtually no binding of RNAP was detected within the same concentration range used for the promoter probes (Fig 3B, probe G3). Under the low-salt condition employed, RNAP forms dimers, which are dissociated after binding to DNA [37,38]. Upon increase of RNAP concentration, super-shift of RNAP-riRNA promoter complex was observed, suggesting RNAP dimerization on DNA templates. However, non-specific binding of RNAP to the probe can’t be ruled out because RNAP often binds to termini of linear DNA. The binding affinity of riRNA promoter was as high as that of the major promoter probe containing both P1 and P2 promoters, indicating that the affinity of RpoD holoenzyme alone in the absence of supporting transcription factors is almost the same between the riRNA promoter and the major promoter. The activity of major promoter is, however, markedly enhanced in the presence of Fis and iNTP and after interaction with the UP element [6,39].

**Table 1:** Probes used in this study.

Probe	Experiment	Size	Region	Position	Comment
**Probe-G1**	Gel shift assay	557	*rrnD* promoter	3429044–3428511	rrnD operon promoter
**Probe-G2**	Gel shift assay	540	*rrsD* (3')	3427532–3426993	riRNA promoter region
**Probe-G3**	Gel shift assay	554	*rrlD* (3')	3426084–3426638	*rrlD* gene
**Probe-A1**	AFM observation	540	*rrsD*	3427532–3426993	riPNA promoter
**Probe-A2**	AFM observation	678	*rrlD*	3424391–3423735	*rrlE* segment
**Probe-T1**	Transcription assay	2366	*rrsD*(3'*)-gltV-rrlD*(5')	3466770–3469167	RS1161/RS1073
**Probe-T2**	Transcription assay		T7 promoter*-gltV-rrlD*(5')		RS83/RS1073
**Probe-R1**	Reporter assay	640	*rrsD*	3428534–3427895	5' proximal *rrsD*
**Probe-R2**	Reporter assay	502	*rrsD*	3427949–3427448	*rrsD* center
**Probe-R3**	Reporter assay	540	*rrsD*	3427532–3426993	3' proximal *rrsD*
**Probe-R3-01**	Reporter assay	140	*rrsD*	3427532–3427393	riRNA promoter segment
**Probe-R3-02**	Reporter assay	240	*rrsD*	3427532–3427293	riRNA promoter segment
**Probe-R3-03**	Reporter assay	340	*rrsD*	3427532–3427193	riRNA promoter segment
**Probe-R3-05**	Reporter assay	127	*rrsD*	3427419–3427293	riRNA promoter segment
**Probe-R3-06**	Reporter assay	134	*rrsD*	3427326–3427193	riRNA promoter segment
**Probe-R3-07**	Reporter assay	138	*rrsD*	3427230–3427093	riRNA promoter segment
**Probe-R3-08**	Reporter assay	137	*rrsD*	3427129–3426993	riRNA promoter segment
**Probe-R3-09**	Reporter assay	238	*rrsD*	3427230–3426993	riRNA promoter segment
**Probe-R3-10**	Reporter assay	334	*rrsD*	3427326–3426993	riRNA promoter segment
**Probe-R3-11**	Reporter assay	427	*rrsD*	3427419–3426993	riRNA promoter segment
**Probe-R4**	Reporter assay	350	*gltV*	3426990–3426639	*rrsD*-*rrlD* spacer
**Probe-R5**	Reporter assay	878	*rrlD*	3426638–3425759	5' proximal *rrlD*
**Probe-R6**	Reporter assay	767	*rrlD*	3425826–3425055	*rrlD* center
**Probe-R7**	Reporter assay	793	*rrlD*	3425147–3424353	*rrlD* center
**Probe-R8**	Reporter assay	679	*rrlD*	3424413–3423735	3' proximal *rrlD*
**Probe-N01**	Northern analysis	640	*rrsD*	3428534–3427895	5' proximal *rrsD*
**Probe-N02**	Northern analysis	502	*rrsD*	3427949–3427448	rrsD center
**Probe-N03**	Northern analysis	540	*rrsD*	3427532–3426993	3' proximal *rrsD*
**Probe-N04**	Northern analysis	326	*gltV*	3426941–3426616	rrsD-rrlD spacer
**Probe-N05**	Northern analysis	216	*gltV*	3426831–3426616	rrsD-rrlD spacer
**Probe-N07**	Northern analysis	878	*rrlD*	3426638–3425759	5' proximal *rrlD*
**Probe-N08**	Northern analysis	762	*rrlD*	3425826–3425055	rrlD center
**Probe-N09**	Northern analysis	793	*rrlD*	3425147–3424353	rrlD center
**Probe-N10**	Northern analysis	679	*rrlD*	3424413–3423735	3' proximal *rrlD*
**Probe-NA1**	Northern analysis	424	*ileT-alaT*	3599565–3599140	*rrnA* operon
**Probe-NA2**	Northern analysis	206	*ileT-alaT*	3599345–3599140	*rrnA* operon

Probes used in the experiment, shown in the second column, contain the sequences of *E*. *coli* K-12 W3110 genome as indicated in the fifth column.

### Binding *in vitro* of RNAP RpoD holoenzyme to riRNA promoter: AFM observation

**Fig 4:**
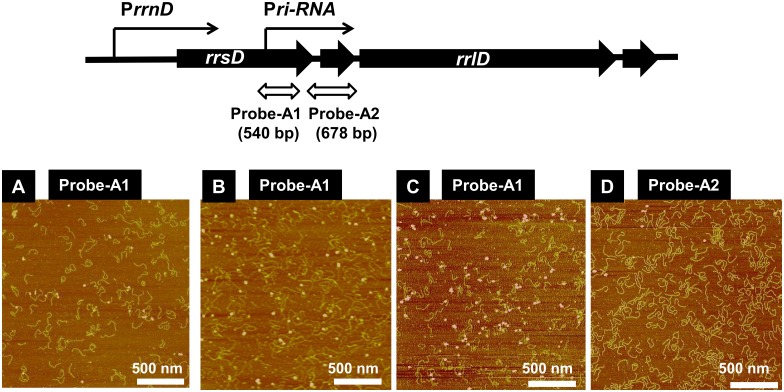
AFM observation of RNAP holoenzyme binding to the riRNA promoter. AFM observation of the RpoD holoenzyme binding to the riRNA promoter was performed following the method as described in Materials and Medhods using two forms of the *rrnE* operon segment, probe A1 (540 bp) and probe A2 (678 bp) (see Table 1), which were prepared by PCR using a set of primers (S1 Table). The amount of DNA probes added were 1 pmol; and the amount of RNAP RpoD holoenzyme was 1 pmol (panel A), 3 pmol (panel B), 10 pmol (panel C) and 1 pmol (panel D).

In order to directly observe the complex formation between riRNA promoter and RNAP RpoD holoenzyme, we employed AFM using a 540 bp-long probe containing the riRNA probe (Table 1, probe A1). Protein concentration-dependent binding of a single molecule of RpoD holoenzyme was observed in the middle of this riRNA probe (Fig 4A–4C). The position of RNAP binding along this riRNA probe was apparently the same between different images, indicating the binding of RNAP at one specific position within this probe. As a reference, we also analyzed a 678 bp-long DNA probe including the spacer region between 16S and 23S rRNA genes of the *rrnE* operon (Table 1, probe A2). Even at the highest concentration of RpoD holoenzme (RNAP:DNA probe = 1:4), no complex formation was observed (Fig 4D). This finding further supports the specific binding of RNAP RpoD holoenzyme to a specific position near the 3’ terminal proximal region of 16S rRNA gene within the *rrnD* operon. The distance of RNAP binding from both ends of this riRNA probe agrees well with the location of riRNA promoter.

### Transcription *in vitro* from the riRNA promoter

**Fig 5:**
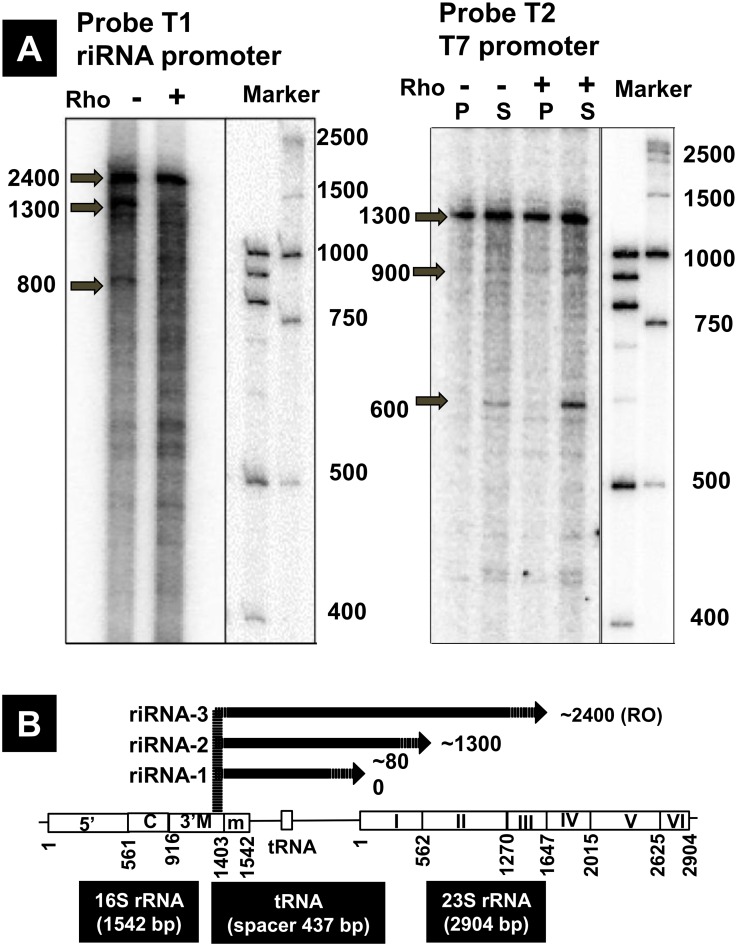
riRNA promoter-directed transcription *in vitro*. [A] Multiple-round transcription *in vitro* was performed using template I (Table 1, probe T1) in the absence and presence of 50 nM Rho (lanes 1 and 2). In the absence of termination factor Rho, three run-off products were indicated, of which the size was estimated to be about 2,400 nt (template-sized run-off transcript), 1,300 nt and 800 nt, respectively. In the presence of Rho, termination was detected at various positions besides the three Rho-independent terminations. Radio-labelled DNA markers are shown to calculate the transcripts size (marker lanes). [B] Transcription in vitro was performed using T7A1 promoter template II (Table 1, probe T2). Transcription was performed in the absence (lanes 1 and 2) and presence (lanes 3 and 4) of 50 nM Rho. The DNA template was 5’ biotinylated and was immobilized on Streptavidin-coated magnetic beads to assess the released transcripts. S and P denote the supernatant and pellet fractions, respectively. Discrete-sized transcripts were detected in the supernatant fraction including released transcripts.

Transcription initiation from the riRNA promoter was further confirmed using *in vitro* transcription system. Template of about 2,400 bp in length containing the riRNA promoter was prepared by PCR and used for *in vitro* transcription by purified RNAP RpoD holoenzyme (Table 1, probe T1). In the absence of termination factor Rho, three major transcripts were detected in multi-round assay (Fig 5A, left panel). The longest transcript of about 2,400 bp in length represents read-through transcript until the template end. In addition, two intermediate sized transcripts were detected, one major transcript of approximately 800 b and another minor transcript of about 1,300 b (Fig 5B). These two transcripts represent Rho-independent termination products, one near the end of spacer and another within 23S rRNA gene near the boundary between 23S rRNA helix-I and -II sequence. This finding indicates that at least half of transcription initiated from the riRNA promoter is terminated prior to the 23S rRNA gene, thereby releasing RNA of approximately 800 b in length including tRNA^Glu^ (Fig 5B). In the presence of Rho, transcription *in vitro* appeared to be terminated at various positions, yielding a number of RNA bands but the level of read-through transcript decreased.

For confirmation of the termination sites, we also performed *in vitro* transcription using the well-characterized strong T7A1 promoter (Fig 5A, right panel). In this case, the transcript of about 1,300 b is pronounced in the absence and presence of Rho. In addition, two smaller transcripts, one 900 b-long RNA and another 600 b-long RNA, were identified. In the presence of Rho, the level of these smaller transcripts increased. This Rho-dependent termination site is located between the tRNA^Glu^ gene but before the 23S rRNA gene (Fig 5B).

We searched for the intrinsic terminal terminator and the Rho-dependent termination signals using ARNold web server (http://rna.igmors.u-psud.fr/toolbox/arnold/) and nFold web server (http://unafold.rna.albany.edu/?q=mfold), respectively. In the region downstream from the tRNA^Glu^ gene to the 5’-proximal region of the 23S rRNA gene, however, we failed to detect a meaning signal of the intrinsic terminator. As to the Rho-dependent terminator, several candidates of low-level signal were identified around the boundary between the spacer and the 23S rRNA gene. Accordingly the level of small transcripts increased in the presence of Rho (see Fig 5A, T7 promoter panel). The absence of strong terminators should be reasonable for the continued transcription of entire *rrn* operons, leading to produce the precursor RNA of rRNA-tRNA composite operons.

### Transcription *in vivo* from the riRNA promoter: Reporter assay

**Fig 6:**
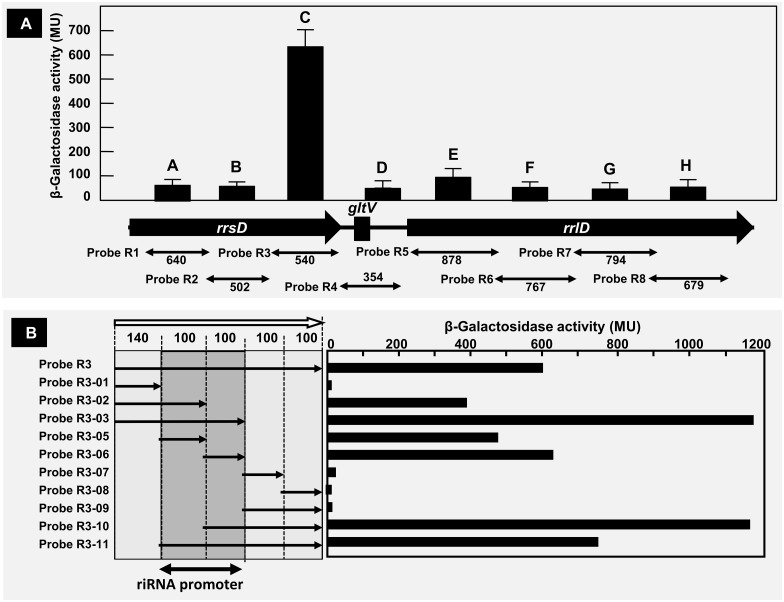
Reporter assay of riRNA promoter. Reporter assay was carried out using *lacZ* reporter gene and a set of *rrnE* operon DNA segments. [A] Eight segments, A to H, of 354~878 bp in length (corresponding to probes R1 to R8 in Table 1), were PCR-amplified from the *rrnD* operon using specific primer pairs (for the primer sequences see S2 Table). PCR-amplified *rrnE* segments were fused to the *lacZ* reporter vector pRS551. The transformants were grown in LB medium and the LacZ activity was measured in the exponential growth phase. High-level expression of LacZ was observed for the reporter vector containing 540 bp-long fragment C (probe 3) that carries the riRNA promoter. [B] For detailed mapping of the riRNA promoter, the 540 bp-long fragment C (probe 3) was divided into 5 sections (5’-proximal 140 bp segment and four 100 bp-long downstream segments). Using these 5 segments, a total of 11 probes (Table 1, R3-01 to R3-11) were synthesized using the respective primers (see S2 Table) and inserted into the LacZ expression vector pRS551. The measurement of LacZ activity was as in [A].

For detection of the promoter activity *in vivo* of this riRNA promoter, we performed a series of reporter assay using LacZ as a reporter. First a set of eight DNA segments (A to H) ranging from 354 to 878 bp in length was prepared from the entire *rrnD* operon and used for construction of the LacZ reporter assay vectors (Table 1, probe R1 to R8). A high-level of LacZ reporter activity was detected only for segment C (probe R3) that corresponds to 3’-terminal proximal region of the 16S rRNA gene (Fig 6A). This finding agrees well with the location of riRNA promoter detected by the gel shift assay and the AFM observation as noted above (see Figs 3 and 4).

In order to further focus the location of riRNA promoter, this 540 bp-long probe R3 (segment C) showing the highest activity of riRNA promoter was divided into 5 pieces, *i*.*e*, one 140 bp-long 5’-proximal segment (probe R3-01 in Table 1) and four 100 bp-long segments (probes R3-05, R3-06, R3-07 and R3-08 in Table 1) (Fig 6B). Using these five minimum segments, a total of ten LacZ reporter assay vectors were constructed in different combinations (Table 1, probe R3-01 to R3-11). Two 100 bp-long minimum segments, probe R3-04 and probe R3-10, exhibited high level of LacZ activity (Fig 6B). Taken the results altogether we concluded the riRNA promoter is located within a narrow region of 200 bp in length between 3427419 and 3427193 on the genome of *E*. *coli* W3110. This region agrees with the binding site of RNAP RpoD holoenzyme as identified by the genomic SELEX screening. The constitutive promoter that is recognized by RpoD holoenzyme alone carries the ideal promoter sequence, TTGACA-(17 bp)-TATAAT [34]. In the center of this 200 bp-long segment, TTACGA-(17 bp)-TACAAT sequence exists, which is located in the junction between R3-04 and R3-10, both exhibiting the highest level of promoter activity.

### Transcription *in vivo* from the riRNA promoter: Northern blot assay

**Fig 7:**
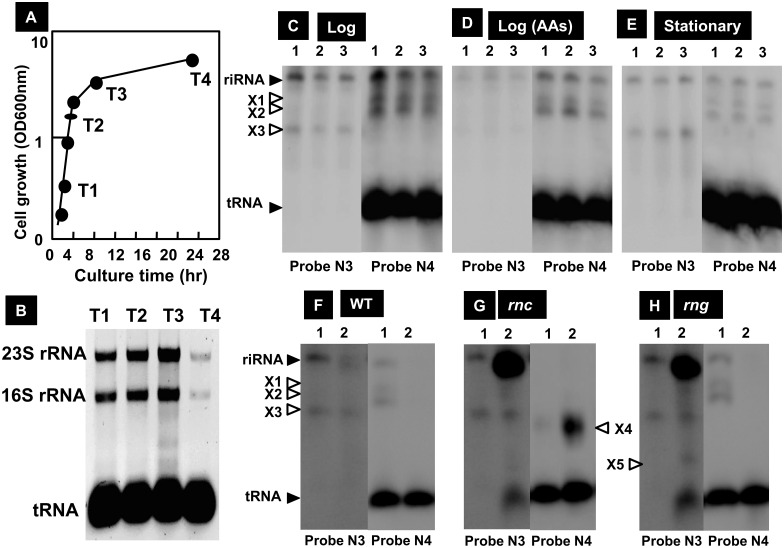
Northern blot analysis of riRNA. [A] Wild-type *E*. *coli* K-12 BW25113 was grown at 37°C in LB medium. Time course of cell growth was monitored by measuring the turbidity at 600 nm. At the indicated times (T1 to T4), cells were harvested and total RNA was prepared using Isogen, a cocktail of phenol plus guanidine thicyanate (Nippon Gene, Japan). [B] The levels of rRNA and tRNA were measured by staining with Ethidium bromide. The level of rRNA markedly decreased in the stationary phase. [C] *E*. *coli* BW25113 was grown in M9-glucose medium, and in the middle of exponential phase, total RNA was prepared using Isogen and subjected to Northern blot analysis using a series of Northern probes (Table 1, probes N01 to N10). riRNA was detected using two specific probes, probe-N3 and probe-N4 (for probes see Table 1; and for primers used for amplification of these probes, see S2 Table). RNA of approximately 800 nt in size was predicted to represent riRNA because it hybridized with both probe-N3 and probe-N4. Besides riRNA band, three predicted processing intermediates of riRNA (X1, X2 and X3) were identified. [D] *E*. *coli* BW25113 was grown in M9 medium supplemented with a complete mixture of 20 amino acids. Total RNA was prepared in the middle of exponential phase and subjected to Northern blot analysis using probe N3 (left panel) or probe N4 (right panel). [E] *E*. *coli* BW25113 was grown in M9-glucose medium and total RNA was prepared in the stationary phase and subjected to Northern blot analysis using probe-N3 (left panel) or probe-N4 (right panel). [F] *E*. *coli* MC1061 (wild-type parent of RNase mutants) was grown in medium E supplemented with casamino acids and glucose. Total RNA was prepared at exponential growth phase (lane 1) and stationary phase (lane 2), and subjected to Northern blot analysis using probe N3 and N4, respectively. Three processing intermediates (X1, X2 and X3) were detected besides riRNA. [G] Total RNA was prepared from *E*. *coli* GM50 (MC1061*rnc*) and subjected to Northern blot analysis as in panel D. The level of riRNA in stationary phase (lane 2) increased compared with wild-type parent. Using probe-N4, a new processing intermediate X4 was detected, that is slightly smaller than X3. [H] Total RNA was prepared from *E*. *coli* GM11 (MC1061 *rng*) and subjected to Northern blot analysis as in panel F. The level of riRNA in stationary phase (lane 2) was higher than that of wild-type parent. Besides the three processing intermediates (X1, X2 and X3), an additional intermediate X5 of smaller size was detected using probe N3.

Next we tried to identify the riRNA *in vivo* using Northern blot analysis. Wild-type *E*. *coli* K-12 W3110 was grown in LB medium (Fig 7A) and at the indicated times (T1 to T4), cells were harvested and total RNA was prepared using Isogen, a cocktail of phenol plus guanidine thiocyanate (Nippon Gene, Japan). The levels of rRNA and tRNA measured by staining with Ethidium bromide were high in exponentially growing phase (Fig 7B). However, the level of rRNA markedly decreased in the stationary phase. Total RNA was subjected to Northern blot analysis using a set of eleven probes (Table 1, probes N01 to N11) covering the entire region of the *rrnD* operon.

Possible riRNA could be identified as those that hybridize with probe N3 corresponding to the riRNA gene but not with upstream probes N1 and N2 of the 16S rRNA gene (see Table 1 for these probes). One specific band of about 800 b in length was detected with use of only two probes, 560 bp-long probe N3 corresponding to the 3’-terminal proximal sequence of the 16S rRNA gene (Fig 7C, lanes 1 to 3) and 326 bp-long probe N4 covering the spacer region (Fig 7C, lanes 4 to 6). Since this band was not detected with use of other nine probes, we predicted this RNA represents riRNA (the primary transcript initiated from the riRNA promoter). Since the sequence including N3 and N4 probes is highly conserved between four type-A *rrn* operons, the predicted riRNA might be originated from four type-A *rrn* operons, *i*.*e*., the *rrnB*, *rrnC*, *rrnD* and *rrnG* operons. When wild-type *E*. *coli* was grown in a rich medium containing a complete mixture of 20 amino acids, the level of riRNA decreased significantly (Fig 7D), implying that the riRNA promoter is not functional when transcription from the major promoters was active, preventing the entry of RNAP to the riRNA promoter.

Besides this riRNA of about 800 b in length, three intermediate bands were detected, *i*.*e*., band X1 and X2 with probe N4, and band X3 with probe N3 (Fig 7C). Since processing intermediates of these sized have not been identified in the pathway of precursor RNA of the entire *rrn* operons [31,32], these bands were predicted to be processing intermediates of riRNA. In the stationary phase, the amounts of riRNA and these processing intermediates decreased (Fig 7E), but this reduction is attributable to the decrease of total RNA recovery in the stationary phase. After correction of the input RNA level, the amount of riRNA relative to the total RNA increased in the stationary phase (Fig 7A). The level of riRNA in exponential phase was estimated to be less than 5% the level of rRNA while it increased to more than 10% in the stationary phase.

Primary transcript of each type-A *rrn* operon of approximately 5,200 b in length includes 16S, 23S and 5S rRNAs and tRNA^Glu^. These precursor composite RNAs are processed through multiple steps into individual mature 16S, 23S and 5S rRNAs and tRNA^Glu^ by the combined actions of more than seven ribonucleases, including RNase III, RNase E, RNase G, RNase R, RNase T, PNPase ad YbeY [40–43]. To examine whether riRNA is processed to produce mature tRNA^Glu^, attempts were made to identify unprocessed intermediates of riRNA in mutants defective in each of these processing nucleases. To test this possibility, Northern blot analysis was performed for RNA samples obtained from various mutants lacking hitherto known processing nucleases. Processing intermediates of riRNA were detected for the mutant lacking RNse III (Fig 7G) and RNase G (Fig 7H). In addition to the three intermediates, X1, X2 and X3, detected in the wild-type parent (Fig 7F), another intermediate X4 was identified in the RNase III mutant, and X5 in the RNase G mutant. Since both X4 and X5 are smaller in size than X3, both RNase III and RNase G were predicted to be involved in later steps of processing.

### Growth phase-dependent control of riRNA expression

**Fig 8:**
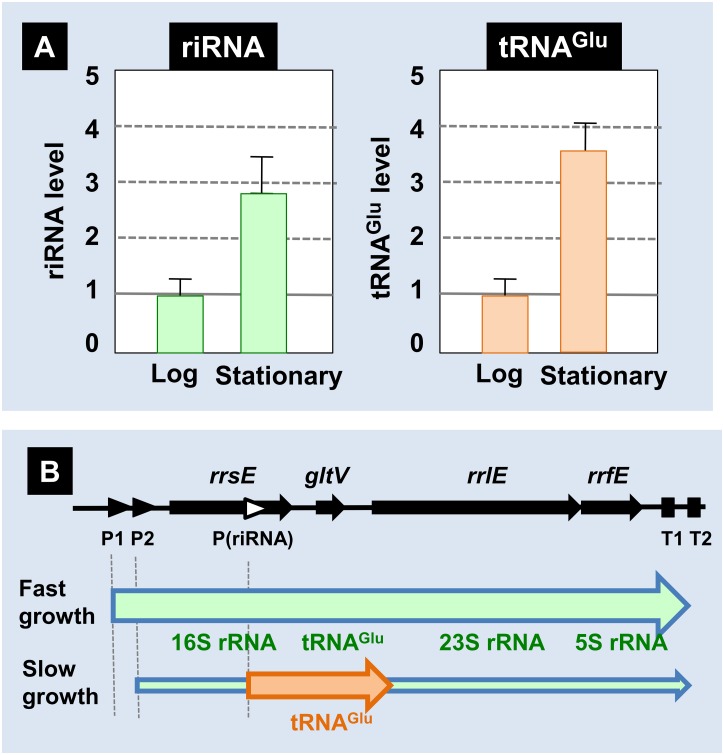
Growth phase-dependent variation of riRNA level. [A] The level of riRNA in *E*. *coli* K12 W3110 grown in M9-glucose medium was determined using probe-N3 and probe-N4 (riRNA panel) as described in Fig 7, while the level of tRNA^Glu^ was determined with use of probe-N4 (tRNA^Glu^ panel). For accurate measurement of riRNA and tRNA, the amount of total RNA applied for Northern blot analysis varied depending on the content of riRNA that altered depending on the culture conditions. The level of riRNA was then determined after correction of total RNA applied. The riRNA level was only a few percent in the exponential growth phase, but increased to more than 10% in the stationary phase while the amount of tRNA^Glu^ was as much as tRNA. By setting the log-phase level at 1, the increase in the stationary phase was determined. [B] On the basis of Northern blot analysis, the levels of both riRNA and tRNA^Glu^ relative to total amount of rRNA was found to increase in the stationary phase. We then propose that the tRNA gene organized within the rRNA-tRNA composite operon is expressed independent of rRNA synthesis under certain specific growth conditions such as in the stationary phase of cell growth.

Based *in vitro* transcription, *in vivo* reporter assays and *in vivo* Northern blot analysis, it is clear that riRNA is synthesized using the internal promoter within the 16S rRNA gene. We next examined the expression level of riRNA under various growth conditions. Total RNA was extracted from *E*. *coli* K-12 W3110 cells grown in M9-glucose medium at various phases of cell growth, and subjected to Nothern blot analysis. A single band of riRNA of about 800 nt in size could be detected by both probe-N03 (3’-proximal 16S rRNA probe as described in Table 1) and probe-N04 (tRNA^Glu^-specific probe as described in Table 1) (see Fig 7C). For quantitative estimation of riRNA, the level of riRNA was normalized based on the total amount of 16S rRNA. The level of riRNA in exponential phase was estimated to be less than 5% the level of rRNA while it increased to more than 10% in the stationary phase. Upon entry into stationary phase, the level increased approximately 3-fold (Fig 8A, riRNA panel).

Using the same RNA samples, we also measured the level of processed tRNA^Glu^ that migrated on PAGE to mature tRNA position and could be detected by using only probe-N03 but not probe-N04 (for the probe list see Table 1). This tRNA^Glu^ could be generated from not only riRNA but also the entire *rrn* operon of all four type-A *rrn* operons, *rrnB*, *rrnC*, *rrnD* and *rrnG* (see Fig 1C). However, the level of tRNA^Glu^ also increased approximately 3.5 fold in the stationary phase (Fig 8B, tRNA^Glu^ panel). These findings indicate that the riRNA promoter is activated in the stationary phase when transcription from the major promoters of *rrn* operons is repressed, thereby allowing the differential synthesis of tRNAs within the rRNA-tRNA composite operons independent of rRNA synthesis under limited conditions.

## Discussion

### Internal promoters within the rRNA-tRNA composite operons

A total of about 4,500 genes on the *E*. *coli* genome are organized into approximately 2,000 operons (average, 2.5 genes per operon), each operon being transcribed into a single polycistronic mRNA [44]. The rRNA-tRNA composite operons of about 5,200 bp in length are typical multi-gene operons consisting of 3 rRNA genes and one to three tRNA genes. Within each of seven *rrn* operons, there are a number of AT-rich promoter-like sequences and several protein-coding capacities of continuous codons. In fact, more than 60% of RNAP RpoD holoenzyme-binding sites on the *E*. *coli* genome were located within open reading frames as identified by Genomic SELEX screening [34]. Within the *E*. *coli rrn* operons, several internal promoters have been proposed in addition to the two major promoters [18,45–49]. After mapping RNAP-binding sites within the *E*. *coli rrnB* sequences carried by lambda transducing phage, RNAP-binding was first notified inside 16S rRNA gene of the *rrnB* operon [45]. Two internal promoters, P16 and P23, were identified inside the 16S rRNA and 23S rRNA genes, respectively [18]. Within the spacer between 16S and 23S rRNA genes of the *rrnB* operon, a weak promoter Pi with the activity 7% the level of *lac* promoter was detected [48]. Transcription from this Pi promoter, however, does not proceed down into the 23S rRNA genes, and thus its functions remains unidentified.

In parallel with the prediction of internal promoters within seven *rrn* operons in *E*. *coli* K-12, the protein-coding possibility has often been proposed for rRNA. For instance, the presence of a 252-bp (84 codon) open reading frame, designated ORF16, was identified within 16S rRNA genes [50] while an ORF consisting of a pentapeptide was identified within the 23S rRNA genes [51]. Overproduction of this mini-gene was found to convert *E*. *coli* cells resistant to the ribosome-inhibiting antibiotic erythromycin [52], suggesting that this E peptide binds to ribosome and modify its function as in the case of peptide antibiotics [53]. Both ORF16 and E-peptide were, however, expressed only when these putative genes were forced to express under the direct control of fused strong promoters. Thus, physiological roles of these putative proteins remain unclear. Along this line, the riRNA promoter herein identified is the first experimentally confirmed internal promoter within the rRNA-tRNA composite operons, which plays a role in expression of tRNA independent of rRNA synthesis.

### Regulatory roles of internal promoters

In past, the regulatory roles of internal promoters have been identified only for a limited number of operons encoding a set of enzymes involved in the same metabolic pathway. For instance, the *E*. *coli trp* operon consists of six genes, *trpLEDCBA*, encoding five enzymes involved in the pathway of Trp synthesis from Glu. A constitutive low-efficiency promoter site was identified in the second structural gene, *trpD* [54]. Transcription *in vitro* is initiated from this internal promoter in the presence of RNAP alone, leading to expression of the downstream *trpC*, *trpB* and *trpA* genes [55]. This internal promoter functions *in vivo* at a level of about 15% of the primary *trp* promoter, but contributes about 80% the level of synthesis of three distal genes under conditions of full repression of the operon [56]. Likewise, within the *ilvLXGMEDA* operon involved in the synthesis pathway of branched amino acids, ILV (Ile, Leu and Val), the major promoter is located upstream *ilvL* encoding a leader peptide [51,52 57,58], but two internal promoters, *ilvE*p and *ilvD*p, exist, each encoding the downstream genes, *IlvEDA* and *ilvDA*, respectively [59,60].

A cell-wall and cell-division operon consists of a total of 16 genes from *mraZ* (the master regulator of this operon for cell division and cell wall genes) and *lpxC* (a lipid biosynthesis gene). The promoter-proximal genes are involved in cell-wall biosynthesis while the promoter-distal genes are required for cell division. In this long operon includes more than 10 internal promoters that allows expression of individual genes using internal promoters [61]. Furthermore, the main promoter-distal cell division genes are regulated by SdiA, the master regulator of cell division [62,63]. After the development of RNA-Seq system, the presence of internal promoters is being widely recognized *in vivo* for a number of long-sized operons. For example, the longest operon so far identified in *E*. *coli* K-12 includes a total of 15 genes in 15,940 bp-long DNA segment [33]. A total of 8 internal promoters were identified within the long complex operon including 15 genes from *nnr* (NAD(P)H-hydrate repair enzyme) to *rlmB* (23S rRNA 2'-*O*-ribose G2251 methyltransferase) [33]. In conjunction with 4 internal terminators, this long-sized operon is predicted to produce a total of 23 different transcripts.

In parallel with the finding of internal promoters by RNA-Seq analysis *in vivo*, the presence of promoter-like sequences within operons has been identified by genomic SELEX analysis *in vitro*. For instance, a total of 2,700 binding sites were identified on the *E*. *coli* K-12 genome for RNAP RpoD holoenzyme, but the number of constitutive promoters recognized by RNAP alone was at most 670, implying the presence of many promoter-like sequences within a set of *E*. *coli* operons [34].

### Differential expression of tRNA from rRNA within the same operons

The expression of transcription and translation apparatus is generally coordinated so as to make the stoichiometric balance in gene expression pathway. To achieve the coordinated expression of components for RNA polymerase and ribosomes, the genes encoding RNA polymerase subunits and ribosomal proteins form the same composite operons in the *E*. *coli* genome, such as RNAP RpoA operon [*rpsM* (S13)-*rpsK* (S11)-*rpsD* (S4)-*rpoA* (RNAP RpoA subunit)-*rplQ* (L17), RNAP RpoBC operon [*rplK* (L11)-*rplA* (L1)-*rplJ* (L10)-*rplL* (L11)-*rpoB* (RNAP RpoB subunit)-*rpoC* (RNAP RpoC subunit)], RNAP RpoZ operon [*rpoZ* (RNAP RpoZ subunit)-*spoT* (ppGpp pyrophosphatase)-*trmH* (tRNA methyltransferase)-*recG* (DNA helicase)] and RNAP RpoD operon [*rpsU* (S21)-*dnaG* (primosome)-*rpoD* (RNAP RpoD subunit)]. These composite operons include the genes encoding components of the core machineries for replication, transcription and translation, thereby making coordination in their intracellular level. The intracellular levels of tRNAs in *E*. *coli* are also maintained in coordination with the levels of ribosomes, and the ratio of tRNA to ribosomes decreases with the increasing growth rate [64]. Approximately 5,200 nt-long transcript of the *rrnE* operon is transcribed into a single RNA, which is then processed into 16S, 23S and 5S rRNA and tRNA^Glu^ by a combination of seven nucleases.

The concentration of tRNA pool is altered over growth range to adjust to the altered codon usage at different growth rates [65–67]. The codon usage in *E*. *coli*, however, differs between genes that are preferentially expressed at high and slow growth rates [68,69]. The genes that are preferentially expressed at fast growth rates are strongly biased toward using the major codons. The composition of the tRNA pool responds to the codon usage in the sense that the relative concentration of some minor tRNA species decreases with increasing growth rates while major tRNA species increases [64,67].

## Materials and Methods

### Bacterial strains

The set of *E*. *coli* K-12 strains used in this study is listed in Table 2. *E*. *coli* K-12 W3110 type-A with the intact *rpoS* gene [70] was used throughout this study, including the purification of RNA polymerase, the construction of an *E*. *coli* genome DNA library for the Genomic SELEX screening, mapping i*n vitro* of riRNA promoters by gel shift assay and AFM observation, reporter assay *in vivo* of riRNA promoters, and Northern blot analysis *in vivo* of riRNA. *E*. *coli* DH5α was used as the host of gene cloning while *E*. *coli* BL21(DE3) was used for expression and purification of the RpoD sigma subunit. *E*. *coli* BW25113 (W3110 *lacI*q *rrnBT14 lacZWJ16 hsdR514 araBADAH33 rhaBADLD78*) [65,71] and JW3229 (a *fis* single-gene deletion mutant of BW25113) [72] were obtained from the *E*. *coli* Stock Center (National Bio-Resource Center, Mishima, Japan). A set of *E*. *coli* mutants defective in RNases for rRNA processing were as follows: *E*. *coli* CM2100 (MG1655 *rne*::*cat* carrying pBD-RNE) [73]; *E*. *coli* GM11 (MG1061 *rng*::*cat*), *E*. *coli* GM20 (MG1061 *rne*-1 *zff*3139:Tn10 Tc); *E*. *coli* GM50 (MG1061 *rnc*-105 *zff*3139:Tn10 Km) [74]. Cells were cultured in Luria-Bertani (LB) or M9-glucose or minimal A-glucose media at 37°C [75]. Cell growth was monitored by measuring the turbidity at 600 nm.

**Table 2:** *Escherichia coli* strains used in this study.

*Escherichia coli* strains used in this study		
Strain	Genotype	Ref
***Escherichia coli* W3110 type-A**	F- IN *(rrnD-rrnE*) *rph-1 rpoS+*	[70]
***Escherichia coli* DH5**	F- *glnX44(AS) deoR481 rfbC1 gyrA96 recA1 endA1 thiE1 hsdR17*	[76]
***Escherichia coli* BL21(DE3)**	*lon-11 (ompT-nfrA)885 (galM-ybhJ) DE3 [lacI lacUV5-T7 gene 1 ind1 sam7 nin5] hsdS10*	[77]
***Escherichia coli* BW25113**	W3110 *lacI*q *rrnBT14 lacZWJ16 hsdR514 araBADAH33 rhaBADLD78*	[71]
***Escherichia coli* JW3229**	BW25113 *fis*	[72]
***Escherichia coli* MG1655**	*ilvG rfb-50 rph-1 fnr-267 eut*	[1]
***Escherichia coli* CM2100**	MG1655 *rme*::*cat* (pBAD-RNE)	[78]
***Escherichia coli* MC1061**	*araD139 (araABC-leu)7698 (lac)X74 galU galK hsdR rpsL150 thi*	[79]
***Escherichia coli* GM11**	MC1061 *rng*::*cat*	[74]
***Escherichia coli* GM20**	MC1061 *rne*::*cat*	[74]
***Escherichia coli* GM50**	MC1061 *rnc-105 zff3139*::*Tn10kan*	[74]

### SELEX search for RNAP RpoD holoenzyme-binding sequences

The genomic SELEX system was used as described previously [35]. For the genomic SELEX screening of recognition sequences of RpoD sigma subunit, RNAP holoenzyme fully saturated with RpoD was reconstituted from the purified core enzyme free from any sigma subunit by adding four molar excess of the purified RpoD protein [34]. The core enzyme free from any sigma subunit was purified as described [36]. After incubation of a mixture of 5 pmol of *E*. *coli* genome DNA fragments, and 10 pmol of RNAP RpoD holoenzyme for 30 min in a binding buffer [10 mM Tris-HCl, pH 7.8 at 4°C, 3 mM Mg acetate, 150 mM NaCl, and 0.01 mg/ml bovine serum albumin], DNA-RNAP complexes were isolated by using anti-RpoC antibody. This SELEX cycle was repeated four rounds and the sequence analysis of RNAP-bound DNA fragments was performed by SELEX-chip (tilling array) method.

### Gel mobility shift assay

Gel mobility shift assay was performed as described previously [80,81]. A set of probes (G1, G2 and G3) (see the probe list in Table 1), each covering a different segment of the *rrnE* operon, were synthesized by PCR using a set of primers (S2 Table), *E*. *coli* K-12 W3110 genome DNA as a template, and Ex *Taq* DNA polymerase (TaKaRa). PCR products were purified using the QIAquick PCR purification kit (Qiagen). For gel shift assays, each of the probes (10 nM) was incubated at 37°C for 30 min with various amounts of RNAP in the gel-shift buffer (50 mM Tris-HCl, pH 7.8 at 37°C, 50 mM NaCl, 3 mM Mg acetate, 0.1 mM EDTA, 0.1 mM DTT, and 0.37 mM BSA). The mixture was directly subjected to 4% PAGE. DNA probes were visualized by staining with ethidium bromide or SYBR Green.

### Atomic force microscopy (AFM) observation

AFM observation of DNA–protein complexes was performed essentially as described [82] using two forms of the *rrnE* operon segment, probe A1 and probe A2 (for the probe list see Table 1), which were prepared by PCR using a set of primers (S2 Table). DNA–protein mixtures were incubated in the binding buffer for 30 min at 37°C, and after cross-linking of DNA–protein by treatment with 0.05% glutaraldehyde for 30 min at 4°C, the samples were diluted with the dilution buffer and directly spotted onto a freshly cleaved mica substrate pretreated with 10 mM spermidine. Imaging was performed in Tapping Mode using a Multimode AFM (Veeco) operation using a Nanoscope IIIa controller. For scanning, Olympus silicon cantilevers (OMCL-AC160TS-W2) with spring constants between 36 and 75 N m21 were used. Image analysis was performed using Nanoscope software.

### Transcription *in vitro*

Linear DNA templates (probes T1 and T2 shown in Table 1) for *in vitro* transcription assays were synthesized by PCR amplification using *E*. *coli* genomic DNA as template and using a set of oligo pairs RS1161/RS1073 and RS83/RS1073 (see S2 Table for the sequences). In order to immobilize the DNA templates to the Streptavidin-coated magnetic beads, a biotin moiety was incorporated to the 5’-end of the templates by using the biotinylated primer RS83 (for the sequence see S2 Table). For detection of riRNA promoter-directed transcription *in vitro*, multiple-rounds of transcription were performed using template-I (probe T1 in Table 1) and in the absence of rifampicin [83]. Reactions were carried out in transcription buffer (20 mM Tris-Cl, pH 8.0, 10 mM MgCl_2_, 50 mM KCl, 1 mM DTT and 100 mg/ml BSA) at 37°C. RNA synthesis is initiated by adding 0.25 mM each of ATP, GTP, UTP and 0.05 mM ^32^P-CTP (3000Ci/mmol). The reactions were stopped and RNA products were isolated by phenol extraction followed by ethanol precipitation. Samples were mixed with 0.01 ml of formaldehyde loading buffer and loaded onto a 6% sequencing gel and analyzed using FLA 9000 Phosphoimager (GE Healthcare).

For detection of termination, transcription was performed using template-II (probe T2 in Table 1) containing the well-characterized T7A1 promoter. The 5’-biotinylated DNA templates were immobilized onto Streptavidin-coated magnetic beads (Promega). The transcription reactions were carried out as described above. Samples were separated into supernatant (S) and pellet (P) fractions by putting the microcentrifuge tubes onto magnetic stands. Half of the supernatant (S) was aspirated out into another microfuge tube. This fraction and the rest of the sample (S+P) were subjected to phenol extraction followed by ethanol precipitation. Samples were mixed with 0.010 ml of formaldehyde loading buffer and loaded onto a 6% sequencing gel and analyzed using FLA 9000 Phosphoimager (GE Healthcare).

### Reporter assay

To measure the activity and regulation of promoters, a LacZ reporter assay was employed by using plasmid vector pRS551 [80,81]. Probes used for the construction of a set of reporter assay vectors (probes R1 to R8; and probes R3-01 to R3-11) were PCR-amplified using *E*. *coli* W3110 genome DNA as a template and sets of specific primers (for the sequence see S2 Table). After digestion with *BamHI* and *EcoRI* [note that the restriction enzyme sites were added in the respective primers], these reporter assay probes (see Table 1 for the location of these probes in the *rrnD* operon) were ligated into pRS551 for construction of a series of the reporter assay plasmids. The construction of the plasmids was confirmed by DNA sequencing. Each plasmid was transformed into *E*. *coli* wild-type K-12 BW25113. Cultures grown overnight in LB medium were diluted 1:1,000 into fresh medium, and cells were grown for 4 h to an optical density at 600 nm (OD600) of 0.4 to 0.5. The activity of LacZ was measured according to the standard Miller method. The measurement was performed three times to obtain average values. Reporter assay was performed in triplicate. The values were indicated using the average of three independent colonies with standard deviation.

### Northern blot analysis

Total RNA was isolated using Isogen, a cocktail of phenol plus guanidine thicyanate (Nippon Gene, Japan). RNA purity was checked by electrophoresis on 2% agarose gel in the presence of formaldehyde followed by staining with Ethidium bromide. Northern blot analysis was performed essentially as described previously [81,84]. Fluorescent-labeled probes, N01 to N10, and the reference probes, NA1 and NA2 (for the probe list see Table 1), were prepared by PCR amplification using 100 ng each of the probe fragments as templates, dNTP substrate mixture including DIG-11-dUTP (Roche), *rrnE* operon region-specific reverse primers (see S2 Table for the sequences), and Ex Taq DNA polymerase (Takara). Total RNAs (0.05 mg) were incubated in formaldehyde-MOPS (morpholinepropanesulfonic acid) gel-loading buffer for 10 min at 65°C for denaturation. RNA samples were directly subjected to electrophoresis on formaldehyde-containing 2% agarose gel, or fractionated on 5% denaturing (8 M urea) polyacrylamide gels in TBE buffer. The RNA was transferred to nylon membrane (Roche) and covalently linked by UV irradiation. Hybridization was performed with DIG easy Hyb system (Roche) at 50°C over night with a DIG-labeled probe. All membranes were washed twice with 2×SSC/0.1% SDS at room temperature for 5 min followed by two 15 min washes with 0.1×SSC/0.1% SDS at 50°C. For detection the DIG-labelled probe, the membrane was treated with anti-DIG-AP Fab fragments and CDP-Star (Roche), and the image was scanned with LAS-4000 IR multi colour (GE Healthcare). Northern blot analysis was performed in triplicate.

## Conclusion

*Escherichia coli* K-12 contains seven copies of the rRNA-tRNA composite operon, each consisting of 16S rRNA-tRNA(s)-23S rRNA-5S rRNA-tRNAs in this order. Using the Genomic SELEX screening, a constitutive promoter was identified within the 16S rRNA gene that was recognized by RNAP RpoD holoenzyme alone (Figs 1, 2 and 6). The purified RpoD holoenzyme alone exhibited strong binding activity to this constitutive promoter (Figs 3 and 4) and promoted transcription *in vitro* (Fig 6). Using Northern blot analysis, the major transcript (riRNA) contained the 3’-terminal proximal sequence of 16S rRNA, the spacer tRNA sequence between 16S and 23S rRNA, and short segments around the junction between the spacer and 23S rRNA (Fig 7). Taken together we propose that the spacer tRNA gene within the rRNA-tRNA composite operons is differentially expressed when the major rRNA promoters were repressed (Fig 8).

## Supporting Information

S1 Fig Junction position of the genome inversion between *E*. *coli* K12 W3110 and K12 MG1655. The inversion junctions of *E*. *coli* K12 W3110 genome are located within the *rrnD* and *rrnE* operons.
(PDF)

S2 Fig Sequence difference of the rRNA genes between seven *rrn* operons in *E*. *coli* K12 W3110. The sequence differences of rRNA genes between seven *rrn* operons of *E*. *coli* K12 W3100 are summarized. The numbers of sequence difference from the consensus sequences among seven *rrn* operons are shown in each operon of 16S and 23S rRNA genes. Total numbers of sequence differences are 33 (2.14%) for 16S rRNA and 77 (2.65%) for 23S rRNA.
(PDF)

S3 Fig Sequence difference of the spacer between 16S and 23S rRNA genes among four type-A *rrn* operons. Type-A rrn operons (*rrnB*, *rrnC*, *rrnD* and *rrnG*) contains tRNA^Glu^ gene inside the spacer between 16S and 23S rRNA genes. The 72-bp long sequence of tRNA^Glu^ gene is identical between *gltT* (*rrnB*), *gltU* (*rrnC*), *gltV* (*rrnD*) and *gltW* (*rrnG*). The sequence difference between 16S rRNA and tRNA^Glu^ genes (5’-proximal spacer) and between tRNA^Glu^ and 23S rRNA genes are shown. Sequence of the *rrnD* operon used for preparation of probes in this study are shown in blue while the sequences different from the most conserved sequences are shown in red.
(PDF)

S1 Table Primers used PCR amplification of probes. Probes used for gel shift assay (probe-G series), *in vitro* transcription assay (probe-T series), reporter assay (probe-R series) and Northern blot analysis (probe-N series) (for the list see Table 1) were constructed by PCR-amplification using the pairs of primers listed in this table.
(PDF)

S2 Table Gene organization of four type-A *rrn* operons in *E*. *coli* K12 W3110. The *rrnB*, *rrnC*, *rrnD* and *rrnG* operons contain tRNA^Glu^ gene inside the spacer between 16R and 23S rRNA genes. The position on the genome and the length of each gene or spacer are described.
(PDF)

## Abbreviations

AFM: atomic force microscopy
riRNA: internal RNA of rRNA operon
RNAP: RNA polymerase
SELEX: systematic evolution of ligands by exponential enrichment
