# Supplementary figures and images for: Differential Regulation of rRNA and tRNA Transcription from the rRNA-tRNA Composite Operon in Escherichia coli
Source: PLoS One. 2016 Dec 22;11(12):e0163057. doi: 10.1371/journal.pone.0163057 (PMC5179076; doi:10.1371/journal.pone.0163057)

## Inversion Junction of the Genome between MG1655 and W3110

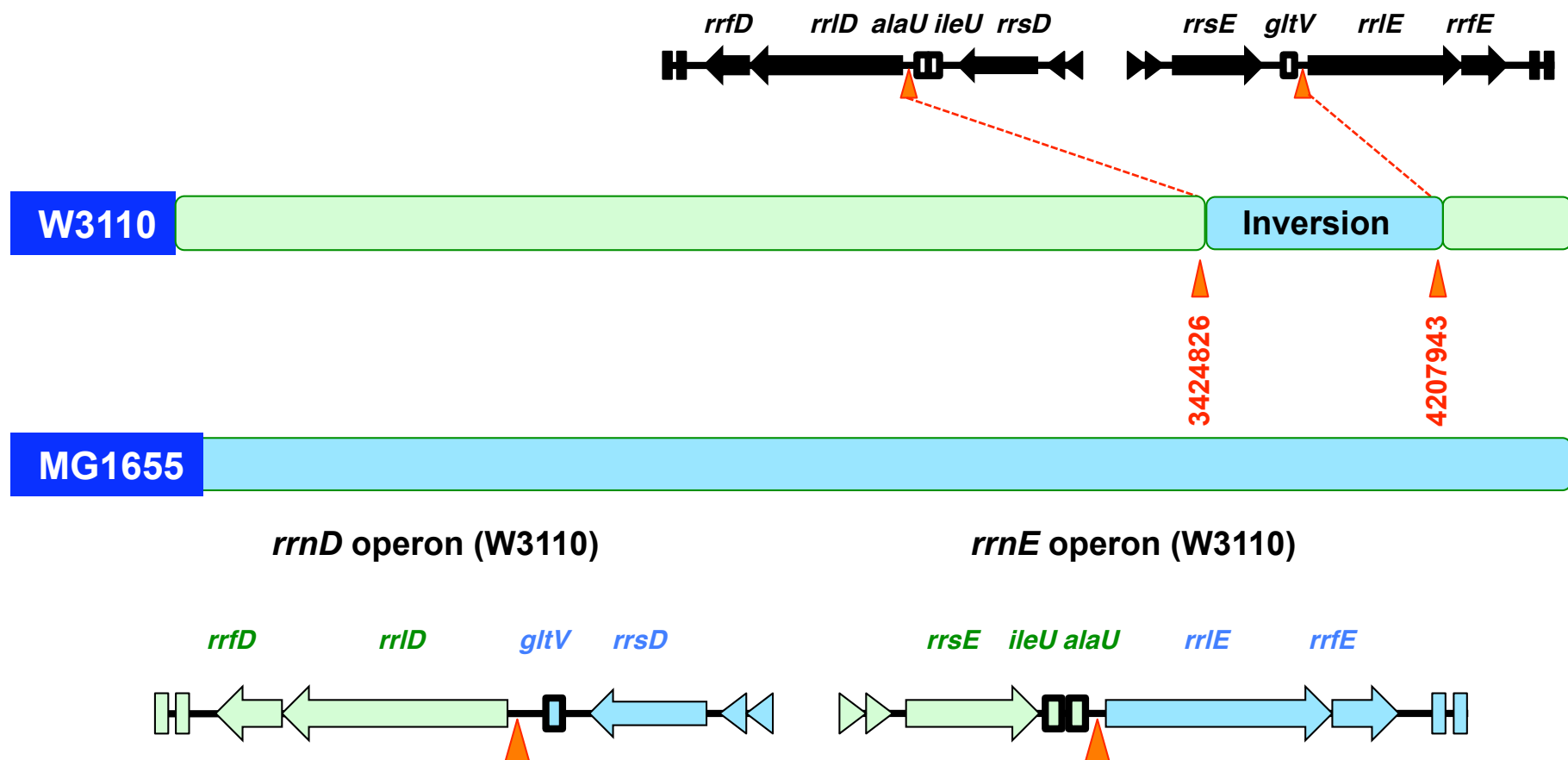

Supplement: S1 Fig — The inversion junctions of E. coli K12 W3110 genome are located within the rrnD and rrnE operons. (PDF) [file pone.0163057.s001.pdf]
