# Supplementary material for: Differential Regulation of rRNA and tRNA Transcription from the rRNA-tRNA Composite Operon in Escherichia coli
Source: PLoS One. 2016 Dec 22;11(12):e0163057. doi: 10.1371/journal.pone.0163057 (PMC5179076; doi:10.1371/journal.pone.0163057)
Supplement: S2 Fig — The sequence differences of rRNA genes between seven rrn operons of E. coli K12 W3100 are summarized. The numbers of sequence difference from the consensus sequences among seven rrn operons are shown in each operon of 16S and 23S rRNA genes. Total numbers of sequence differences are 33 (2.14%) for 16S rRNA and 77 (2.65%) for 23S rRNA. (PDF) [file pone.0163057.s002.pdf]

Sequence Difference of rRNA between Seven *rrn* Operons

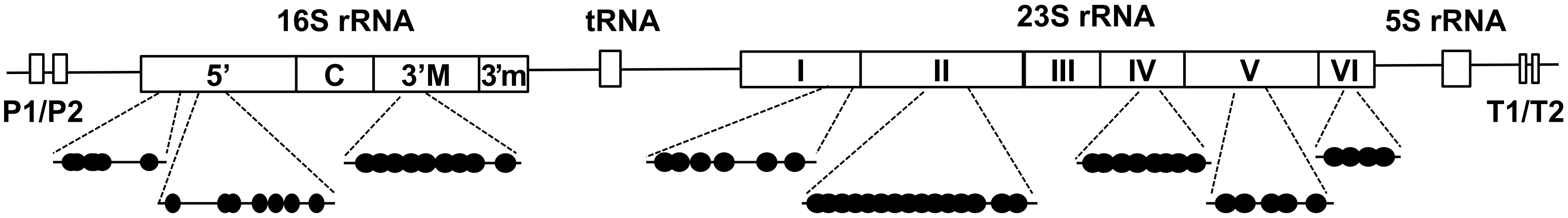

16S rRNA
Total length 1,542
Total difference 33 (2.14%)

rrnA	1
rrnB	0
rrnC	6
rrnD	0
rrnE	9
rrnG	8
rrnH	9

23S rRNA
Total length 2,904
Total difference 77 (2.65%)

rrlA	26
rrlB	12
rrlC	7
rrlD	5
rrlE	9
rrlG	12
rrlH	6
