# Supplementary material for: Differential Regulation of rRNA and tRNA Transcription from the rRNA-tRNA Composite Operon in Escherichia coli
Source: PLoS One. 2016 Dec 22;11(12):e0163057. doi: 10.1371/journal.pone.0163057 (PMC5179076; doi:10.1371/journal.pone.0163057)
Supplement: S3 Fig — Type-A rrn operons (rrnB, rrnC, rrnD and rrnG) contains tRNAGlu gene inside the spacer between 16S and 23S rRNA genes. The 72-bp long sequence of tRNAGlu gene is identical between gltT (rrnB), gltU (rrnC), gltV (rrnD) and gltW (rrnG). The sequence difference between 16S rRNA and tRNAGlu genes (5’-proximal spacer) and between tRNAGlu and 23S rRNA genes are shown. Sequence of the rrnD operon used for preparation of probes in this study are shown in blue while the sequences different from the most conserved sequences are shown in red. (PDF) [file pone.0163057.s003.pdf]

Sequence Difference of Spacers between Type-A *rrn* Operons

5′-Proximal spacer

```
ccttaaagaagcgtactttgtagtgctcacacagattgtctgatagaaagtgaaaagcaa
ccttaaagaagcgttctttgcagtgctcacacagattgtctgataggaagtgaaaagcaa
ccttaaagaagcgttctttgcagtgctcacacagattgtctgatagaaagtgaaaagcaa
ccttaaagaagcgtactttgcagtgctcacacagattgtctgatgaaaagtgaatagcaa

ggcgtttacgcgttgggagtgaggc (rrnB)
ggcgtcttgcgaagcagactgatac (rrnC)
ggcgtcttgcgaagcagactgatac (rrnD)
ggcgtttacgcgttgggagtgaggc (rrnG)
```

3′-Proximal spacer

```
cttgctggtttgtgagtgaaagtcgccgaccttaatatctcaaaactcatcttcgggtga
cttgctggtttgtgagtgaaagtcacctgccttaatatctcaaaactcatcttcgggtga
cttgctggtttgtgagtgaaagtcacctgccttaatatctcaaaactcatcttcgggtga
cttgctggtttgtgagtgaaagtcgccgacctcaatatctcaaaacagactgttaagtct

tgtttgagatatttgctctttaaaaatctggatcaagctgaaaattgaaacactgaacaa
tgtttgagatatttgctctttaaaaatctggatcaagctgaaaattgaaacactgaacaa
tgtttgagatatttgctctttaaaaatctggatcaagctgaaaattgaaacactgaacaa
tgtttgatatatttgctctttaaaaatctggatcaagctgaaaattgaaacactgaacaa

cgagagttgttcgtgagtctctcaaattttcgcaacacgatgatgaatcgaaagaaacat
cgaaagttgttcgtgagtctctcaaattttcgcaacacgatgatgaatcgaaagaaacat
cgaaagttgttcgtgagtctctcaaattttcgcaacacgatgatgaatcgtaagaaacat
tgaaagttgttcgtgagtctctcaaattttcgcaactctgaagtgaaacat----------

cttcgggttgtga  (rrnB)
cttcgggttgtga  (rrnC)
cttcgggttgtga  (rrnD)
cttcgggttgtga  (rrnG)
```
