# Supplementary material for: Differential Regulation of rRNA and tRNA Transcription from the rRNA-tRNA Composite Operon in Escherichia coli
Source: PLoS One. 2016 Dec 22;11(12):e0163057. doi: 10.1371/journal.pone.0163057 (PMC5179076; doi:10.1371/journal.pone.0163057)
Supplement: S1 Table — Probes used for gel shift assay (probe-G series), in vitro transcription assay (probe-T series), reporter assay (probe-R series) and Northern blot analysis (probe-N series) (for the list see Table 1) were constructed by PCR-amplification using the pairs of primers listed in this table. (PDF) [file pone.0163057.s004.pdf]

S1 Table
Sequence of four *rrn* operons (W3110)
(*rrnB, rrnC, rrnD, rrnG*)

	16S rRNA	5' Spacer	tRNA^{Glu}	3' Spacer	23S rRNA
rrnB	3,468,481 - 3,470,022	3,468,039 - 3,468,480 (440 bp)			3,465,137 - 3,468,040
	3,470,022 > 3,468,481	3,468,480 > 3,468,310	3,468,309 > 3,468,234	3,468,233 > 3,468,041	3,468,040 > 3,465,137
	**1,542**	**171**	**76 (*gltT*)**	**193**	**2,904**
	**1,542**	**440**			**2,904**
rrnC	3,693,332 - 3,694,873	3,692,976 - 3,693,331 (354 bp)			3,690,074 - 3,692,977
	3,694,873 > 3,693,332	3,693,331 > 3,693,247	3,693,246-3,693,171	3,693,170-3,692,978	3,692,977 > 3,690,074
	**1,542**	**85**	**76 (*gltU*)**	**193**	**2,904**
	**1,542**	**354**			**2,904**
rrnD	3,426,993 - 3,428,534	3.426,992 > 3,426,639 (353 bp)			3,423,735 - 3,426,639
	3,428,534 > 3,426,993	3.426,992 > 3,426,908	3,426,907-3,426,832	3,426,831-3,426,640	3,426,639 > 3,423,735
	**1,542**	**85**	**76 (*gltV*)**	**192**	**2,904**
	**1,542**	**353**			**2,904**
rrnG	2,728,272 - 2,729,813	2,728,271 > 2,727,841 (431 bp)			2,727,937 - 2,727,840
	2,729,813 > 2,728,272	2,728,271 > 2,728,101	2,728,100 > 2,728,025	2,728,024 > 2,727,841	2,727,840 > 2,724,937
	**1,542**	**171**	**76 (*gltW*)**	**184**	**2,904**
	**1,542**	**431**			**2,904**
