# Supplementary material for: Differential Regulation of rRNA and tRNA Transcription from the rRNA-tRNA Composite Operon in Escherichia coli
Source: PLoS One. 2016 Dec 22;11(12):e0163057. doi: 10.1371/journal.pone.0163057 (PMC5179076; doi:10.1371/journal.pone.0163057)
Supplement: S2 Table — The rrnB, rrnC, rrnD and rrnG operons contain tRNAGlu gene inside the spacer between 16R and 23S rRNA genes. The position on the genome and the length of each gene or spacer are described. (PDF) [file pone.0163057.s005.pdf]

S2 Table
Primers used for PCR amplification of probes

Probe	Primer	Sequence
**Probe-G1**	PrrD-P-F	CATCCTTATTTTTCGCCCGCATTG
	PrrD-P-R	AGCCATGATCAAACTCTTCAATTTAAAAG
**Probe-G2**	PriRNA-F	GAAGTTTTCAGAGATGAGAATGTGCC
	PriRNA-G	TAAGGAGGTGATCCAACCGCAG
**Probe-A1**	PrrsD-3-F	CGCGGATCCGAAGTTTTCAGAGATGAGAATGTGCC
	PrrsD-3-R	CGCGGATCCTAAGGAGGTGATCCAACCGCAG
**Probe-A2**	PrrlD-4-F	CGCGGATCCCAGTGTCTGGTGGGTAGTTTGAC
	PrrlD-4-R	CGCGGATCCAAGGTTAAGCCTCACGGTTCATTAG
**Probe-T1**	RS1073	GGGCTTTTCACCCGCTTTATC
	RS1161	AGGTTGTGCCCTTGAGGCGTGGCTTC
**Probe-T2**	RS83	CCATAAACTGCCAGGAATTGGGGAT
	RS1073	AGCGGGCTTTTCACCCGCTTTATC
**Probe-R1**	PrrsD-1-F	CGCGGATCCAAATTGAAGAGTTTGATCATGGCTCAG
	PrrsD-1-R	CGCGGATCCTCAGATGCAGTTCCCAGGTTGAG
**Probe-R2**	PrrsD-2-F	CGCGGATCCCGGTTTGTTAAGTCAGATGTGAAATC
	PrrsD-2-R	CGCGGATCCCAACATTTCACAACACGAGCTGAC
**Probe-R3**	PrrsD-3-F	CGCGGATCCGAAGTTTTCAGAGATGAGAATGTGCC
	PrrsD-3-R	CGCGGATCCTAAGGAGGTGATCCAACCGCAG
**Probe-R3-01**	PrrsD-3-F	CGCGGATCCGAAGTTTTCAGAGATGAGAATGTGCC
	PrrsD-3-1-R	CGCGGATCCGGCCGGACCGCTGGCAACAAAGGATA
**Probe-R3-02**	PrrsD-3-F	CGCGGATCCGAAGTTTTCAGAGATGAGAATGTGCC
	PrrsD-3-2-R	CGCGGATCCATTGTAGCACGTGTGTAGCCCTGGTCGTAAGG
**Probe-R3-03**	PrrsD-3-F	CGCGGATCCGAAGTTTTCAGAGATGAGAATGTGCC
	PrrsD-3-3-R	CGCGGATCCGATTCCGACTTCATGGAGTCGAGTTGCAGACTCCAATC
**Probe-R3-05**	PrrsD-3-1-F	CGCGGATCCTATCCTTTGTTGCCAGCGGTCCGGCCG
	PrrsD-3-2-R	CGCGGATCCATTGTAGCACGTGTGTAGCCCTGGTCGTAAGG
**Probe-R3-06**	PrrsD-3-2-F	CGCGGATCCTTACGACCAGGGCTACACACGTGCTACAATGG
	PrrsD-3-3-R	CGCGGATCCGATTCCGACTTCATGGAGTCGAGTTGCAGACTCCAATC
**Probe-R3-07**	PrslD-3-3-F	CGCGGATCCGATTGGAGTCTGCAACTCGACTCCATGAAGTCGGAATC
	PrrsD-3-4-R	CGCGGATCCTACCTACTTCTTTTGCAACCCACTCCCATGGTGTGA
**Probe-R3-08**	PrrsD-3-4-F	CGCGGATCCTCACACCATGGGAGTGGGTTGCAAAAGAAGTAGGTAG
	PrrsD-3-R	CGCGGATCCTAAGGAGGTGATCCAACCGCAG
**Probe-R3-09**	PrrsD-3-3-F	CGCGGATCCGATTGGAGTCTGCAACTCGACTCCATGAAGTCGGAATC
	PrrsD-3-R	CGCGGATCCTAAGGAGGTGATCCAACCGCAG
**Probe-R3-10**	PrrsD-3-2-F	CGCGGATCCTTACGACCAGGGCTACACACGTGCTACAATGG
	PrrsD-3-R	CGCGGATCCTAAGGAGGTGATCCAACCGCAG
**Probe-R3-11**	PrrsD-3-1-F	CGCGGATCCTATCCTTTGTTGCCAGCGGTCCGGCCG
	PrrsD-3-R	CGCGGATCCTAAGGAGGTGATCCAACCGCAG
**Probe-R4**	PrrlD-0-F	CGCGGATCCTTAAAGAAGCGTTCTTTGCAGTG
	PrrlD-0-R	CGCGGATCCTCACAACCCGAAGATGTTTCTTAC
**Probe-R5**	PrrlD-1-F	CGCGGATCCGGTTAAGCGACTAAGCGTACACG
	PrrlD-1-R	CGCGGATCCCTTGCCGAAACAGTGCTCTACC
**Probe-R6**	PrrlD-2-F	CGCGGATCCCCGAAAGCTATTTAGGTAGCGCC
	PrrlD-2-R	CGCGGATCCATGCTTAGAGGCTTTTCCTGGAAG
**Probe-R7**	PrrlD-3-F	CGCGGATCCGCAAATCCGGAAAATCAAGGCTG
	PrrlD-3-R	CGCGGATCCCGTGCTCCTCCGTTACTCTTTAG

**Probe-R8**	PrrlD-4-F	CGCGGATCCCAGTGTCTGGTGGGTAGTTTGAC
	PrrlD-4-R	CGCGGATCCAAGGTTAAGCCTCACGGTTCATTAG
**Probe-N01**	PrrsD-1-F	CGCGGATCCAAATTGAAGAGTTTGATCATGGCTCAG
	PrrsD-1-R	CGCGGATCCTCAGATGCAGTTCCCAGGTTGAG
**Probe-N02**	PrrsD-2-F	CGCGGATCCCGGTTTGTTAAGTCAGATGTGAAATC
	PrrsD-2-R	CGCGGATCCCAACATTTCACAACACGAGCTGAC
**Probe-N03**	PrrsD-3-F	CGCGGATCCGAAGTTTTCAGAGATGAGAATGTGCC
	PrrsD-3-R	CGCGGATCCTAAGGAGGTGATCCAACCGCAG
**Probe-N04**	tRNA Probe-1F	GAAAAGCAAGGCGTCTTGCGAAGCAGACTGATAC
	tRNA Probe-1R	CGTGTACGCTTAGTCGCTTAACC
**Probe-N05**	tRNA Probe-2F	CTTGCTGGTTTGTGAGTGAAAGTCACCTGC
	tRNA Probe-1R	CGTGTACGCTTAGTCGCTTAACC
**Probe-N07**	PrrlD-1-F	CGCGGATCCGGTTAAGCGACTAAGCGTACACG
	PrrlD-1-R	CGCGGATCCCTTGCCGAAACAGTGCTCTACC
**Probe-N08**	PrrlD-2-F	CGCGGATCCCCGAAAGCTATTTAGGTAGCGCC
	PrrlD-2-R	CGCGGATCCATGCTTAGAGGCTTTTCCTGGAAG
**Probe-N09**	PrrlD-3-F	CGCGGATCCGCAAATCCGGAAAATCAAGGCTG
	PrrlD-3-R	CGCGGATCCGCAAATCCGGAAAATCAAGGCTG
**Probe-N10**	PrrlD-4-F	CGCGGATCCCAGTGTCTGGTGGGTAGTTTGAC
	PrrlD-4-R	CGCGGATCCAAGGTTAAGCCTCACGGTTCATTAG
**Probe-NA1**	tRNA Probe-3F	TGAAAATGAGCAGTAAAACCTCTACAGGCTTGTAGCTCA
	tRNA Probe-1R	CGTGTACGCTTAGTCGCTTAACC
**Probe-NA2**	tRNA Probe-4F	TCTCTGTAGTGATTAAATAAAAAATACTTC
	tRNA Probe-1R	CGTGTACGCTTAGTCGCTTAACC
